# Copper-Infused MXene from MAX Phase for Enhanced Electrochemical Ammonia Production

**DOI:** 10.1021/acsnano.5c09659

**Published:** 2025-11-12

**Authors:** Radhika Nittoor-Veedu, Bindu Kalleshappa, Martin Pumera

**Affiliations:** † Future Energy and Innovation Laboratory, Central European Institute of Technology, 48274 Brno University of Technology, Purkyňova 123, Brno 61200, Czech Republic; ‡ Quantum Materials Laboratory, 3D Printing and Innovation Hub, Center for Nanorobotics and Machine Intelligence, Department of Chemistry and Biochemistry, Mendel University, Zemědělská 1, Brno 61300, Czech Republic; § Department of Medical Research, China Medical University Hospital, China Medical University, No. 91 Hsueh-Shih Road, Taichung 40402, Taiwan; ∥ Advanced Nanorobots & Multiscale Robotics Laboratory, Faculty of Electrical Engineering and Computer Science, VSB - Technical University of Ostrava, 17. Listopadu 2172/15, Ostrava 70800, Czech Republic; ⊥ Department of Chemical and Biomolecular Engineering, Yonsei University, 50 Yonsei-ro, Seodaemun-gu, Seoul 03722, Korea; # Energy Research institute@NTU (ERI@N), Research Techno Plaza, X-Frontier Block, Level 5, 50 Nanyang Drive, Singapore 637553, Singapore

**Keywords:** 2D materials, doping, catalysis, electrocatalysis, clean energy

## Abstract

The electrochemical conversion of nitrate to value-added ammonia is a green alternative to the energy-intensive Haber–Bosch process. However, due to inefficient catalysts, guiding the reaction route towards selective ammonia synthesis is still challenging. Here, we report a highly efficient Cu-infused V_2_C catalyst, synthesized via the molten salt synthesis method using selective CuCl_2_ etching. The optimized catalyst exhibits a faradaic efficiency of ∼83% and an ammonia yield of 320 μg cm^–2^ h^–1^ at −0.7 V vs RHE. We performed ^15^N isotopic labeling to ensure the purity and origin of ammonium ions. The synthesis approach was further applied to Ti_3_C_2_ MXene, which, upon Cu infusion, achieved a faradaic efficiency of ∼70% and an improved ammonia yield of approximately 450 μg cm^–2^ h^–1^ at −0.6 V vs RHE. Our results demonstrate that Cu-infused MXene catalysts achieve high selectivity, faradaic efficiency, and ammonia yield, highlighting the broad applicability of this method across different MXenes derived from various transition metals. Moreover, the Cu infusion approach can be expanded to incorporate other metals, offering versatile potential for developing MXene-based catalysts for a range of applications, from ammonia synthesis to CO_2_ reduction.

## Graphical abstract

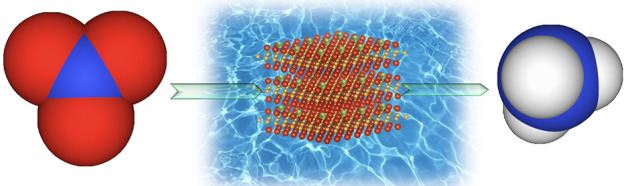

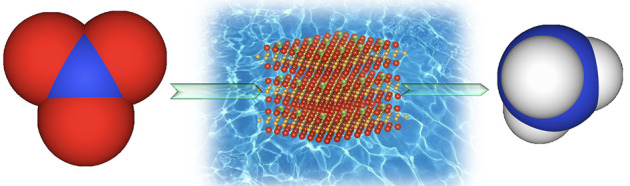

## Introduction

Ammonia has emerged as one of the most extensively manufactured industrial chemicals globally, serving as a cornerstone in agriculture, explosives production, and leather processing.^1,2^ It is also now being reimagined as a key player in the future of clean energy by serving as a carbon-free hydrogen carrier^3^ and sustainable fuel for mobile and remote applications.^4–6^ Traditionally, ammonia is synthesized via the Haber–Bosch process, an energy-intensive method that operates under high pressure and temperature and contributes significantly to global greenhouse gas emissions.^7^ As a more sustainable and environmentally friendly alternative, electrochemical reduction of nitrogen-based compounds has emerged as a promising approach for ammonia production.^7^ Among these, the electrochemical nitrate reduction (NO_3_RR) method offers several advantages, such as utilizing nitrate-contaminated wastewater as a feedstock, thereby repurposing a pollutant while reducing wastewater treatment costs.^8^ Moreover, nitrate reduction is more energy-efficient than direct nitrogen gas conversion, as the N–O bond in nitrate is weaker compared to the strong triple bond in nitrogen gas. However, the widespread adoption of electrochemical ammonia synthesis hinges on the development of highly efficient and durable electrocatalysts to activate the N–O bond and enhance the overall efficiency and cost-effectiveness of the process.

MXene is a recent addition to two-dimensional (2D) materials, with a central transition metal (M) and either carbon, nitrogen, or both (X), and surface functional groups (T_*x*_), including oxygen, fluorine, hydrogen, etc.^9,10^ Distinguished from its parent MAX phase, where an aluminum layer (A) separates metal carbides or nitrides, by enhanced chemical properties, MXene shows promise in various fields, including biomedical applications, sensors, energy storage, etc.^11–13^ Traditionally, MXene synthesis from MAX phases involved etching the aluminum layer using highly toxic hydrofluoric acid (HF), prompting researchers to explore safer alternatives. To mitigate the hazards of HF, MXenes have recently been produced using fluoride-based alternatives, such as LiF, NaF, (NH_4_)­HF_2_, etc., in combination with HCl. However, the aqueous etching process involving F-containing compounds still poses significant toxicity and corrosion risks, hindering the large-scale production of MXenes. To overcome these challenges, fluorine-free MXene synthesis methods have been developed, including electrochemical etching, ball milling, hydrothermal, chemical vapor deposition (CVD), and Photo-Fenton techniques.^14–18^ Despite their potential, scalability remains a major limitation. Recently, etching MAX phases in molten salts with Lewis acid characteristics has emerged as a more efficient approach for synthesizing a wide range of MXenes. This method benefits from the shielding effect of molten salts, enabling high-temperature processing in air while allowing for tunable interlayer spacing and surface functionalization. Notably, Ma et al.^19^ introduced a one-pot molten-salt synthesis method in an air atmosphere, successfully etching the Ti_3_AlC_2_ MAX phase at 700 °C. Inspired by this work, we infused elemental copper (Cu) particles into V_2_CT_*x*_ and Ti_3_C_2_T_*x*_ MXenes during the etching to utilize in catalyzing the ammonia production reaction. Given copper’s well-established catalytic activity in NO_3_RR, its integration within the MXene structure further boosts the material’s efficiency for this reaction.^20^

Here, we developed a catalyst by infusing Cu into MXene layers through a reaction between the MAX phase and CuCl_2_, achieving complete removal of the aluminum (Al) layer while simultaneously embedding Cu nanoparticles within the MXene sheets. The presence of Cu nanoparticles significantly enhances the electrochemical performance for nitrate reduction compared to MXene structures, where Cu is entirely removed. Our approach not only enhances the performance of MXene but also highlights the potential for tailored modifications to optimize its catalytic properties. We successfully synthesized Cu-infused V_2_C MXene, achieving a Faradaic efficiency (FE) of up to 83% and an impressive ammonia yield rate (YR) of 320 μg cm^–2^ h^–1^ at −0.7 V vs RHE, along with enhanced stability for up to ten cycles. Encouraged by these results, we extended this method to other categories of MXene, specifically Ti_3_C_2_ MXene, which also demonstrated a promising Faradaic efficiency of 70% and ammonia yield of 460 μg cm^–2^ h^–1^ at −0.6 V vs RHE. These findings underscore the versatility and broad applicability of Cu-infusion nanoarchitectonics^21^ in enhancing the catalytic performance of diverse MXene materials.

## Results and Discussion

### Physicochemical Characterization of Cu@V_2_C

The Cu-infused MXene was synthesized using Lewis acid (CuCl_2_)-assisted molten-salt synthesis, as shown in the schematic Figure 1A. The scanning electron microscopy (SEM) analysis was conducted to examine the morphology of the synthesized catalyst and to confirm the successful etching of the parental MAX phase into MXene. Figure 1B depicts a block of V_2_AlC MAX phase, characterized by a dense structure due to the strong interlayer bonding. To assess the impact of Cu on ammonia production, the V_2_AlC MAX phase was etched using chloride salts without CuCl_2_, thereby exposing the inner layers, as illustrated in Figure 1C. The synthesis follows a replacement reaction mechanism, where Al in the A layer of MAX is substituted by Cu in the presence of CuCl_2_. However, due to the weak interaction between Cu and the V_2_C layer, Cu ions tend to aggregate into Cu particles. This is evident in Figure 1D, which depicts Cu@V_2_C, with Cu particles trapped between the V_2_C layers after Al etching. This is further supported by the EDS mapping shown in Figure 1E.

**Figure 1:**
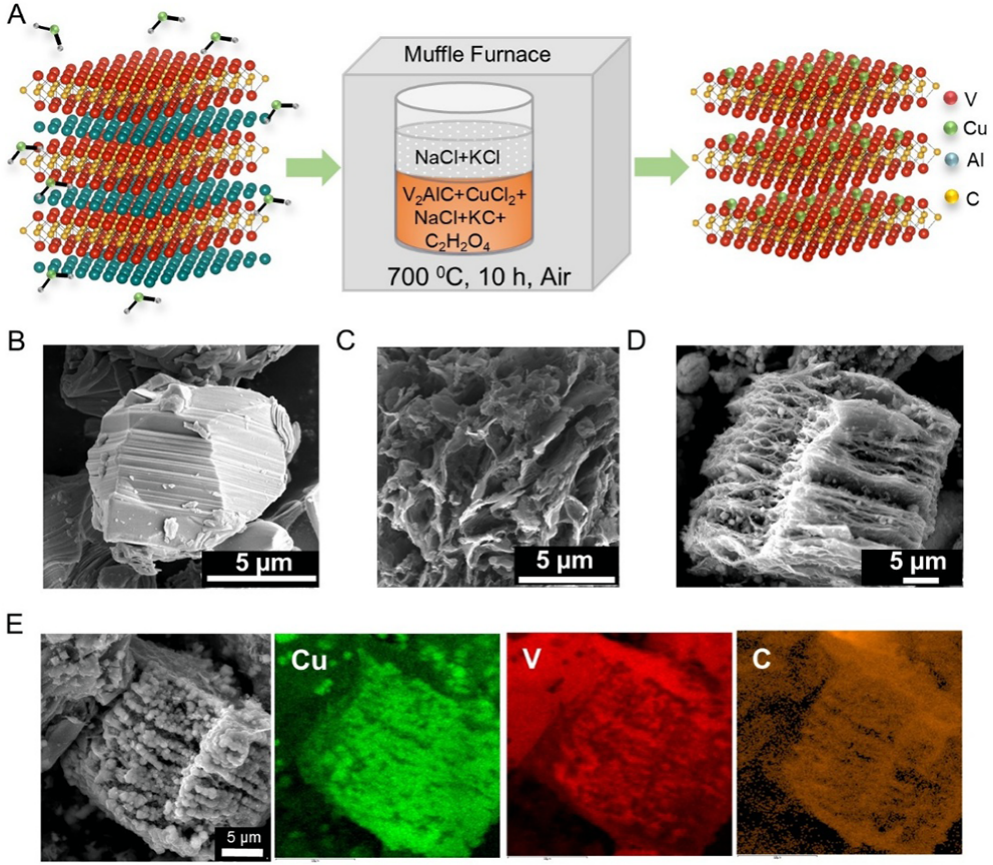
Synthesis and morphological studies of Cu@V_2_C: A) Schematic representation of the molten-salt synthesis method. Scanning electron microscopy images of B) V_2_AlC, C) V_2_CTx, D) Cu@V_2_C. E) Scanning electron microscopy images and corresponding EDS mapping of Cu@V_2_C.

The chemical and structural profiling of MXenes was studied using X-ray diffraction (XRD) and X-ray photoelectron spectroscopy (XPS). The XRD patterns for the V_2_AlC MAX phase and Cu@V_2_C are displayed in Figure 2A­(b) and (c), respectively. The diffraction pattern of the V_2_AlC MAX phase closely matches the ICDD-00-029-0101 reference pattern (Figure 2A­(a)), confirming its hexagonal crystal structure. In Figure 2A­(c), diffraction peaks at 2θ = 36° correspond to the (100) crystallographic plane of V_2_C, whereas the peak at 2θ = 41.26° is attributed to the (103) plane. The characteristic peak for V_2_C around 9° is not prominent in Cu@V_2_C, likely due to the increased interlayer spacing caused by the insertion of Cu particles between the MXene layers.^22,23^ The XRD pattern of Cu@V_2_C shows no additional peaks associated with Al-containing phases, confirming the successful exfoliation of the Al layer. Moreover, distinct peaks corresponding to elemental Cu are prominently observed at 2θ = 43.8°, 50.9°, and 74.4° (marked with a diamond shape), indicating the successful incorporation of Cu within the MXene structure. Additionally, the presence of weak diffraction peaks related to CuO at 37.2 eV suggests the possible formation of CuO during the sample-washing process.

**Figure 2:**
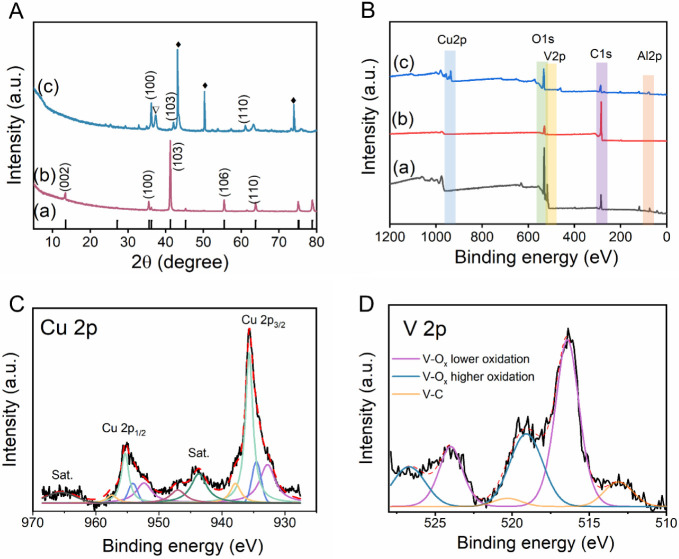
Structural analysis of Cu@V_2_C. (A) XRD patterns of Cu@V_2_C samples; (a) ICDD-00-029-0101, (b) V_2_AlC, (c) Cu@V_2_C. (B) XPS wide spectra of (a) V_2_AlC, (b) V_2_C, and (c) Cu@V_2_C. XPS core-level spectra of (C) Cu 2p and (D) V 2p.

The XPS was carried out to analyze the chemical composition of the material. The XPS survey spectra of Cu@V_2_C (Figure 2B­(c)) reveal the characteristic peak of Cu 2p at a binding energy of approximately 932 eV, proving the successful incorporation of Cu in the MXene. It is noticeable that the Al peak is completely removed in V_2_C (Figure 2B­(b)); however, an insignificant amount of unwashed Al was present in the Cu@V_2_C (Figure 2B­(c)), as chlorides and oxides formed during the synthesis. The core-level spectra were analyzed to better understand the elemental and chemical composition of the synthesized Cu@V_2_C. The Cu 2p spectrum (Figure 2C) was deconvoluted into Cu 2p_1/2_ and Cu 2p_3/2_ components with three satellite peaks located at 943.8, 947.1, and 965.2 eV originating from the divalent Cu species. Each of the Cu 2p_1/2_ and Cu 2p_3/2_ was further fitted into four distinct components, revealing the coexistence of Cu^2+^ and Cu^0^ species. The peak corresponding to Cu^0^ at 935 eV reveals the formation of Cu nanoparticles, clustered on the MXene surface. The primary contribution of Cu^2+^ arises from the presence of oxide (934.6 eV) and chloride (935.7 eV), and a minor contribution from other divalent Cu (937.8 eV), possibly the surface Cu–OH formed during the etching process. The chloride surface groups on the V_2_C MXene layers are attributed to the use of CuCl_2_ as the etchant for the Al layer separation, while the Cu^2+^ oxides formation is likely a result of the oxidation during the subsequent washing steps. Furthermore, the V 2p spectrum (Figure 2D) reveals the formation of V–C at a binding energy of 513.1 eV, corresponding to the development of V_2_C, along with oxides at 516.4 and 519.1 eV, generated as a result of the etching and washing. Additionally, the high-resolution spectra of C 1s and O 1s are depicted in Figure S1. The C 1s spectra show a dominant peak at 284.8 eV, corresponding to the C–C bond, along with signals from various oxygen-containing groups such as C=O, C–O, and COOH, at 286.2, 288.0, and 291.6 eV, respectively, reflecting varying degrees of oxidation during the washing step. The O 1s spectrum displays a prominent peak attributed to V–C–O bonding, alongside signals corresponding to Cu–O species, corroborating the findings from the Cu 2p spectra. Hydroxyl and carbonyl groups are also observed, indicating oxidation of the Cu@V_2_C surface during the synthesis process.^24–27^

### Electrochemical Properties of Cu@V_2_C

Further electrochemical analysis was carried out to assess the potential of Cu@V_2_C as an electrocatalyst for ammonia synthesis. The electrical impedance and charge transport properties of Cu@V_2_C were investigated using electrochemical impedance spectroscopy (EIS) in 0.1 M KNO_3_ + 0.5 M Na_2_SO_4_, a powerful technique for probing electron and ion transport in electrochemical systems. EIS provides insights into fundamental processes such as mass diffusion, adsorption, chemical reactions, and interfacial phenomena. The Nyquist plot of Cu@V_2_C is presented in Figure S2A, showing an incomplete single semicircle with relatively lower impedance. The diameter of the semicircle corresponds to the charge-transfer resistance (*R*_ct_). The Equivalent circuit fitted to *R*_s_ + (*R*_1_|*Q*_1_) + (*R*_2_|*Q*_2_),^28^ and the detailed EIS parameter values obtained from the analysis are provided in Figure S2B.

The linear sweep voltammetry (LSV) was carried out using the Cu@V_2_C as the catalyst in 0.5 M Na_2_SO_4_ and 0.1 M KNO_3_ + 0.5 M Na_2_SO_4_ to study their performance toward NO_3_RR. The experiment is repeated for the V_2_C catalyst as well to compare the performance in the absence of Cu, as shown in Figure 3A. The lower reduction potential using the Cu@V_2_C and its higher difference in the reduction potential in the presence and absence of nitrate ions suggest that Cu@V_2_C possesses certain electrochemical activity toward NO_3_RR. Both the V_2_C and Cu@V_2_C are then electrolyzed for 1 h, applying varying potential from −0.4 to −0.9 V vs RHE using the chronoamperometry (CA) method in an H-cell separated by a glass frit (Figure S3). The H-cell setup helps to reduce the back oxidation at the anode chamber and hence improves the efficiency of the catalyst. During the electrolysis, a higher current density is observed with Cu@V_2_C (Figure 3B) compared to pure V_2_C (Figure S4A), showing that the Cu insertion enhances the electrochemical performance. After the electrolysis, a certain amount of the catholyte was collected from the H-cell to quantify the ammonia produced during the electrolysis, using the colorimetric method. The collected catholyte was diluted to the detection range, and the absorbance was measured using UV–vis spectroscopy.

**Figure 3:**
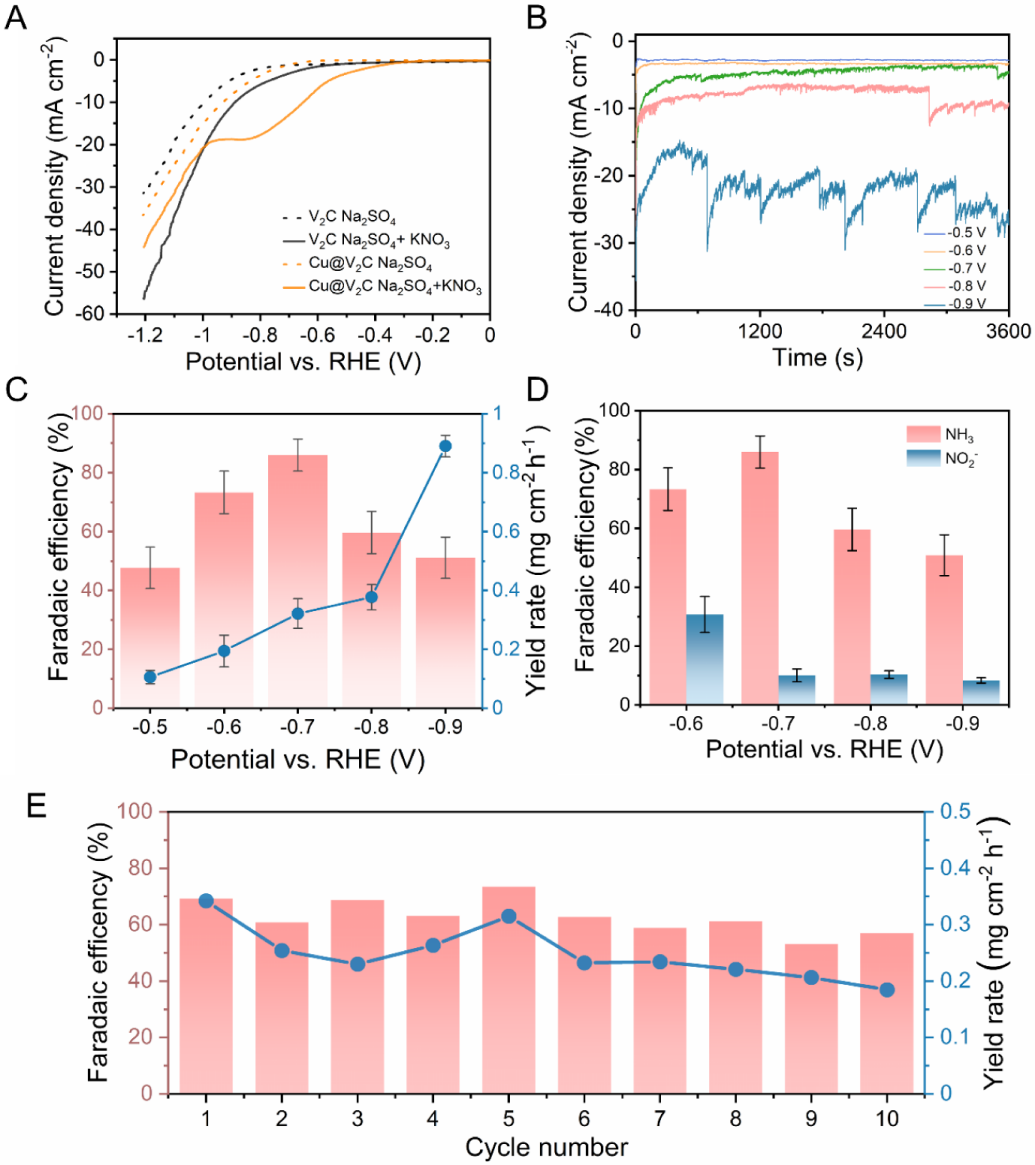
Electrochemical ammonia synthesis: A) Linear sweep voltammetry of V_2_C and Cu@V_2_C in 0.5 M Na_2_SO_4_ (dashed line) and 0.5 M Na_2_SO_4_ + 0.1 M KNO_3_ (solid line). B) Chronoamperometry curves for Cu@V_2_C at different potentials. C) Ammonia yield and FE for Cu@V_2_C at different potentials. D) FE for ammonia and nitrite at different potentials. E) The durability tests of Cu@V_2_C for NO_3_RR at − 0.7 V vs RHE.

As shown in Figure S4B, the electrolyte using V_2_C exhibited weak absorbance at all potentials, consistent with its inert behavior toward NO_3_RR, as indicated by the LSV data in Figure 3A. In contrast, the electrolyte from Cu@V_2_C displayed a distinct absorption peak at 655 nm (Figure S5A) across different applied potentials, confirming the presence of ammonium ions in the solution after electrolysis. The ammonium concentration was quantified using the standard curve in Figure S6 obtained from the absorption of a known concentration of ammonium chloride (NH_4_Cl) solution. The FE and YR are calculated using eqs 2 and 3, respectively, derived from the law of electrolysis. Upon varying the potential, the FE (Figure 3C) initially surged from ∼50% at −0.5 V to ∼83% at −0.7 V vs RHE, followed by a gradual decline at more negative potentials. Meanwhile, the ammonia yield steadily increased from ∼150 μg cm^–2^ h^–1^ to ∼900 μg cm^–2^ h^–1^ with increasing potential. Our previous studies on pure MXenes for NO_3_RR reported an FE of ∼60% for V_2_C at −1.0 V vs RHE.^26^ This highlights the enhanced catalytic performance of Cu@V_2_C at lower reduction potentials, attributed to the synergistic effects between the V_2_C MXene and the interlayer-infused Cu. The superior catalytic performance of Cu@V_2_C relative to V_2_C can be ascribed to its larger electrochemically active surface area (ECSA), as illustrated in Figure S7. Cyclic voltammetry (CV) measurements for V_2_C and Cu@V_2_C are presented in Figure S7A,C, respectively, with the integrated area under the CV curves of Cu@V_2_C exceeding that of V_2_C at the same scan rate. Correspondingly, the slopes of the Δ*j*/2 versus scan rate plots (Figure S7B,D) give ECSA values of 64,190 cm^2^ g^–1^ for Cu@V_2_C and 40,812 cm^2^ g^–1^ for V_2_C.

To determine the selectivity of the catalyst toward ammonia production, the amount of nitrite (NO_2_^–^) byproduct was also determined with the Griess method using UV–vis spectroscopy (Figure S5B). The NO_2_^–^ concentration was determined using a calibration curve derived from standard solutions with known NO_2_^–^ concentrations as shown in Figure S8. The FE for NO_2_^–^ is less than 30% and further decreases with an increase in potentials, as shown in Figure 3D. This confirms the higher selectivity of Cu@V_2_C toward ammonia production. Since the highest FE was marked at −0.7 V vs RHE, this potential was selected for further stability tests. The Cu@V_2_C was tested up to ten cycles, and it remained relatively stable in terms of efficiency and yield over the cycles, demonstrating its potential as an efficient electrocatalyst for NO_3_RR. The post characterization of the catalyst was carried out using SEM. The catalyst, after the extensive 10 cycles, was collected from the glassy carbon working electrode and analyzed. The catalyst sustained its original shape and composition after the intense ten cycles of measurement, as shown in Figure S9. The well-known affinity of Cu toward oxygen-containing species facilitates the initial reduction steps at a reduced reduction potential. Additionally, the higher conductivity of Cu nanoparticles helps in boosting the performance, along with acting as a spacer between V_2_C layers, preventing restacking and maintaining the high surface area for a longer duration, improving the stability of the catalyst.

Further, an isotopic labeling experiment was carried out to ensure the origin of ammonia in the catholyte and to detect any NH_3_ contamination using the nuclear magnetic resonance (NMR) technique performed on ^15^N-labeled NO_3_^–^. The typical double peak (Figure 4A) of ^15^NH_4_^+^ appears in the ^1^H NMR spectra. In contrast, the typical triplet from ^14^NH_4_^+^ was obtained when the ^14^KNO_3_ was used as the source (Figure 4B), which proves that the produced ammonia is completely derived from the electroreduction of NO_3_^–^.

**Figure 4:**
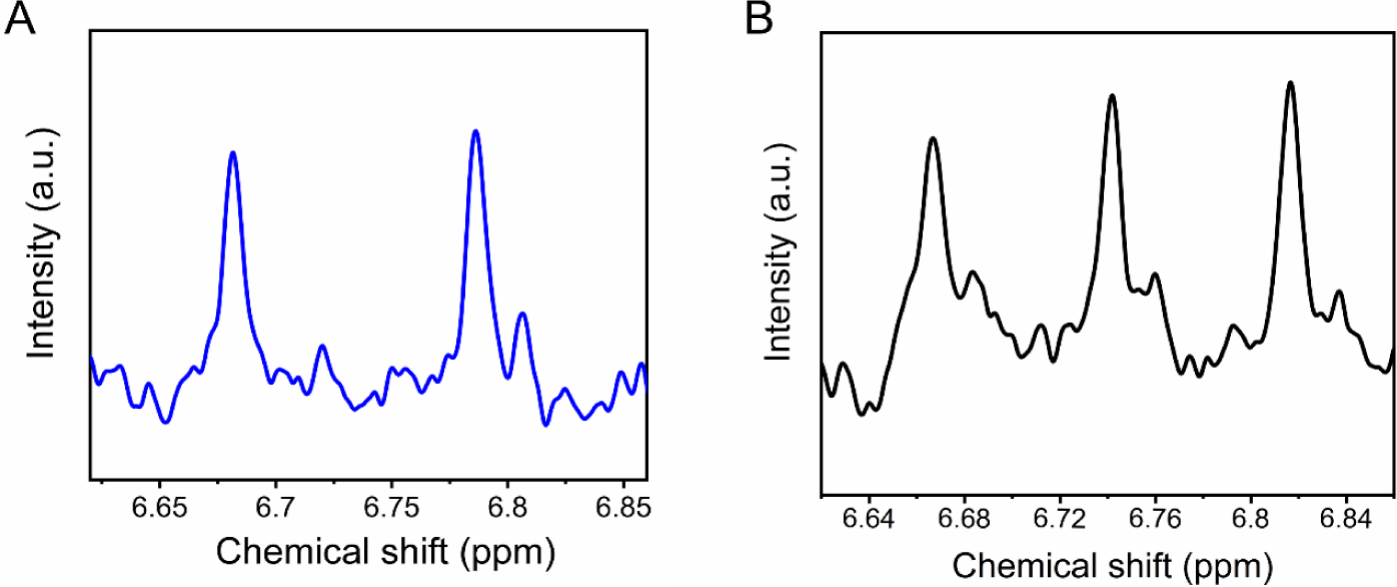
^1^H NMR measurement. A) ^1^H NMR spectra from K^15^NO_3_. B) ^1^H NMR spectra from K^14^NO_3_.

### Structural Analysis and Electrochemical Performance of Cu@Ti_3_C_2_

To assess the applicability of this synthesis method, Cu@Ti_3_C_2_ was synthesized similar to Cu@V_2_C using CuCl_2_ as a Lewis acid etchant in the molten-salt synthesis method. As shown in Figure 5A, the Ti_3_AlC_2_ MAX phase exhibits a block-like structure due to the strong interlayer bonding of Ti, C, and Al layers. The Ti_3_C_2_ MXene etched in the absence of CuCl_2_ is represented in Figure 5B, where Ti_3_C_2_ layers are well separated. Figure 5C shows the morphology of the Cu@Ti_3_C_2_, which indicates the separation of layers after the removal of the Al-layer using CuCl_2_ by the replacement mechanism. Unlike in Cu@V_2_C, Cu is uniformly distributed across the Ti_3_C_2_ layers without agglomerating into nanoparticles, as confirmed by the elemental distribution of Cu@Ti_3_C_2_ using SEM-EDS (Figure S10).

**Figure 5:**
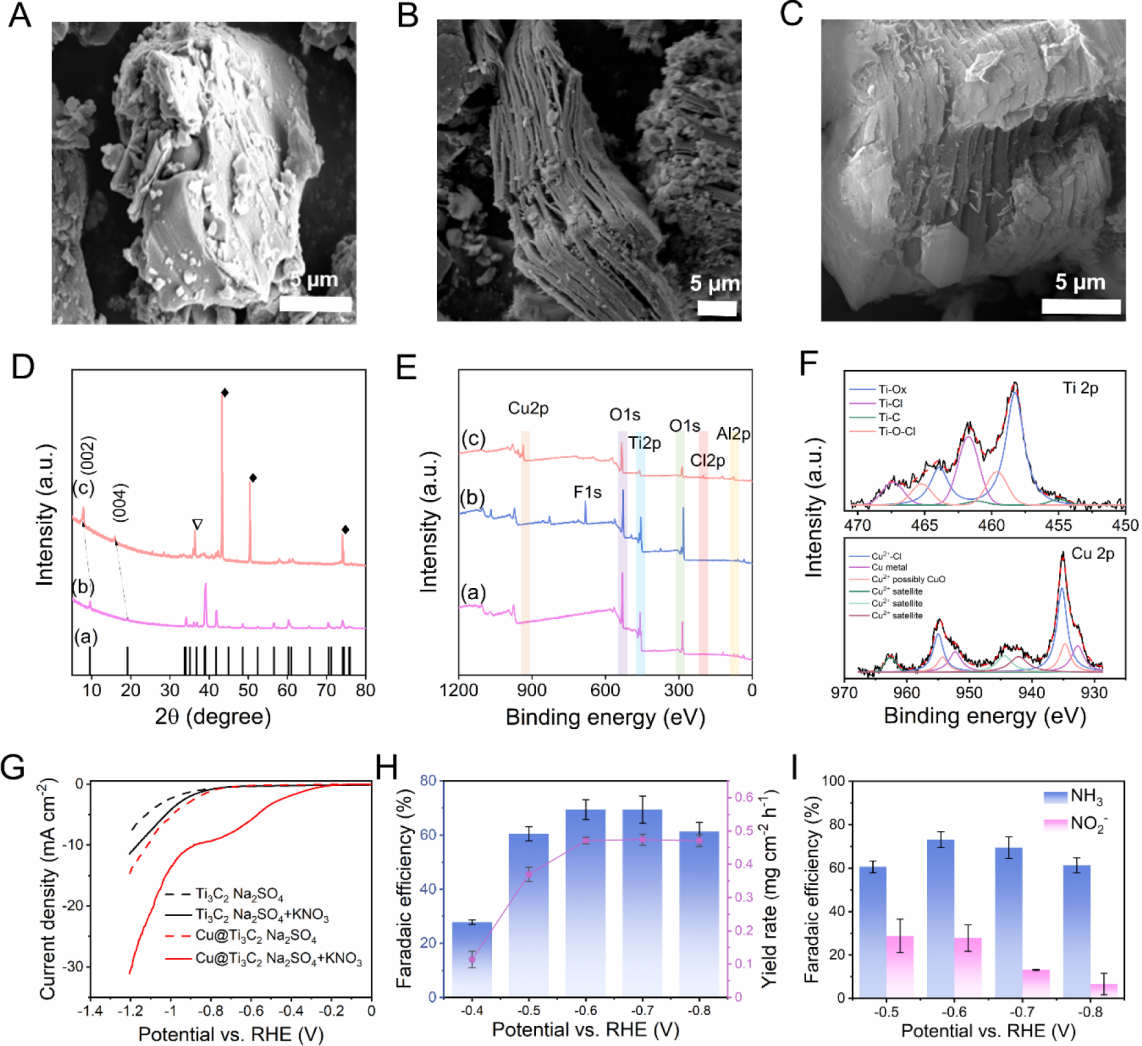
Structural analysis and electrochemical performance of Cu@Ti_3_C_2_. SEM images (5 μm scale bar) of A) Ti_3_AlC_2_, B) Ti_3_C_2_, and C) Cu@Ti_3_C_2_. D) XRD spectra of (a) ICDD, (b) Ti_3_AlC_2_, (c) Cu@Ti_3_C_2_. E) XPS wide spectra of Cu@Ti_3_C_2_ ((a) Ti_3_AlC_2_, (b) Ti_3_C_2_, (c) Cu@Ti_3_C_2_). F) XPS core-level spectra of Ti 2p (upper panel) and Cu 2p (lower panel). G) Linear sweep voltammetry of Ti_3_C_2_ and Cu@Ti_3_C_2_ in 0.5 M Na_2_SO_4_ (dashed line) and 0.5 M Na_2_SO_4_ + 0.1 M KNO_3_ (solid line). H) Ammonia yield and FE for Cu@Ti_3_C_2_ at different potentials. I) FE for ammonia and nitrite synthesis using Cu@Ti_3_C_2_ at different potentials.

The XRD analysis was performed on the Cu@Ti_3_C_2_ catalyst to investigate the crystalline structure, with the corresponding pattern shown in Figure 5D. The Ti_3_AlC_2_ spectra, shown in Figure 5D­(b), align with the ICDD-00-029-0101 reference (Figure 5D­(a)), confirming the hexagonal crystal structure of the Ti_3_AlC_2_ MAX phase. However, unlike V_2_C, the (002) and (004) planes at approximately 2θ = 9° and 13° in Cu@Ti_3_C_2_ are not significantly diminished but instead shifted to a lower 2θ value of 7.9°, confirming the formation of etched Ti_3_C_2_. This shift indicates an increase in the interlayer spacing due to the Al-layer removal and Cu insertion.^29^ The sharp peaks of metallic Cu from (111), (200), and (220) crystal planes, along with minor peaks from Cu oxides,^30^ suggesting the possible formation of CuO, resulting from the sample-washing process. The absence of peaks related to Al from the MAX phase to MXene further confirms the complete etching of the Ti_3_AlC_2_.

Additionally, the XPS analysis of the Cu@Ti_3_C_2_ is carried out to study the chemical composition of the catalyst, as depicted in Figure 5E. The appearance of the Cu peak at 931 eV confirms the successful infusion of Cu particles in the Ti_3_C_2_ matrix. The chemical composition of the material is further studied using high-resolution XPS spectral analysis. As depicted in Figure 5F, the Ti 2p (Figure 5F upper panel) is deconvoluted into four peaks. The Ti–C peak at 455 eV confirms the successful formation of Ti_3_C_2_. A peak at 458.3 eV, corresponding to Ti–O, is attributed to the presence of oxygen terminal groups introduced during the washing process. Additionally, the detection of chloride signals in the high-resolution spectra indicates the formation of −Cl surface terminations, resulting from the CuCl_2_-based etching process. Furthermore, the high-resolution Cu 2p peaks show the elemental Cu species at 932.7 eV along with divalent Cu species, associated with the chlorides (935.2 eV) and oxides (934.8 eV) formed during the etching and subsequent washing process, respectively. The satellite peaks observed at 942.1, 944.4, and 962.7 eV, originating from shakeup processes, also confirm the presence of Cu^2+^ species. Moreover, the C 1s spectrum (Figure S11) displays characteristic peaks corresponding to Ti–C and a significant amount of C–C at 281.5 and 284.8 eV, respectively, further validating the formation of Ti_3_C_2_ after successful etching. Additional peaks at 287.9 and 286.2 eV are attributed to C–O and C–H bonds, respectively, likely introduced via surface functionalization during the washing process.^26,27,31^

For studying the electrochemical performance of the Cu@Ti_3_C_2_ catalyst toward NO_3_RR, the impedance measurement was carried out using 0.1 M KNO_3_ + 0.5 M Na_2_SO_4_, and the Nyquist plot of Cu@Ti_3_C_2_ is shown in Figure S12 with its equivalent circuit and the parameters. Similar to the Cu@V_2_C system, Cu@Ti_3_C_2_ is kinetic-controlled in nature. To evaluate the NO_3_RR performance of Ti_3_C_2_ and Cu@Ti_3_C_2_, the LSV was conducted using the same electrolyte used for the Cu@V_2_C studies (0.5 M Na_2_SO_4_ and 0.1 M KNO_3_ + 0.5 M Na_2_SO_4_). It is evident that Cu@Ti_3_C_2_ exhibits a significantly larger reduction potential difference of approximately −0.4 V in the presence and absence of nitrate ions, whereas Ti_3_C_2_ shows only a subtle increase in reduction potential when used as the catalyst. (Figure 5G). To analyze the electrochemical ammonia production capacity, both Ti_3_C_2_ and Cu@Ti_3_C_2_ are then subjected to 1 h electrolysis in the H-cell using 0.5 M Na_2_SO_4_ + 0.1 M KNO_3_, applying a constant potential as depicted in Figures S13A and S14A, respectively. After the completion of electrolysis, a certain amount of catholyte was collected from the H-cell and subjected to detection and further quantification of ammonia using the colorimetric method. A sharp peak at 655 nm was observed for all the higher potentials using Cu@Ti_3_C_2_, confirming the presence of ammonia (Figure S14B). Whereas the pure Ti_3_C_2_ showed a diminished current density at the same potential and a very low absorption peak by UV–vis spectroscopy, indicating a negligible amount of ammonia (Figure S13B). The enhanced catalytic activity of Cu@Ti_3_C_2_ can be attributed to its higher ECSA, estimated using the same method as for Cu@V_2_C. The CV curves and Δj/2 versus scan rate plots for Ti_3_C_2_ and Cu@Ti_3_C_2_ are presented in Figure S15. The calculated ECSA values are 33,267 cm^2^ g^–1^ for Ti_3_C_2_ and 69,304 cm^2^ g^–1^ for Cu@Ti_3_C_2_, reflecting the significantly greater active surface area of the Cu-modified material.

From the concentration obtained using the UV–vis spectroscopy method, further quantification has been carried out. As depicted in Figure 5H, the FE increases gradually from −0.3 to −0.6 V vs RHE and reaches up to 70%, stabilizes a little in that range of applied potential, and decreases upon further increasing the applied voltage. The ammonia YR also steadily increased from the beginning and stabilized at −0.6 V vs RHE with a YR of ∼450 μg cm^–2^ h^–1^. To study the selectivity of Cu@Ti_3_C_2_ toward ammonia production, the important byproduct of the reaction, NO_2_^–^, is also determined and quantified using the Griess method using UV–vis spectroscopy. As shown in Figure 5I, the FE of the NO_2_^–^ is lower than 30% and further decreases during the higher potentials, showing the selectivity of the catalyst toward ammonia synthesis. The UV–vis absorption spectra for NO_2_^–^ are shown in Figure S16. Since we obtained the maximum FE and a stabilized YR at −0.6 V vs RHE, we selected this potential for testing the catalytic stability (Figure S17). The stability was tested up to six cycles, and the catalyst remained at a roughly similar range of FE and YR without a steep change throughout the cycle. In our previous study,^26^ it was shown that the pure Ti_3_C_2_ has an FE of around 75% at −1.2 V vs RHE; the Cu insertion significantly reduces the reduction potential of the catalyst for NO_3_RR. Even though MXene exhibits good conductivity, the incorporation of Cu nanoparticles further enhances the electron transfer kinetics at the electrode–electrolyte interface, hence boosting the reduction reaction at a lower potential.^32,33^ The affinity of Cu toward oxygen-containing species such as NO_3_^¯^ and other intermediates helps in providing more favorably oriented adsorption sites for these species compared to the pristine MXene. Hence, proving that the catalysts produced using this method are efficient in terms of FE, YR, selectivity, and stability for ammonia production.

## Conclusion

Inspired by the green etching process of MAX phases for MXene synthesis, we investigated a molten salt etching method employing CuCl_2_ to achieve complete removal of the Al-layer while simultaneously embedding Cu nanoparticles between the MXene sheets. The resulting catalysts demonstrated a significant efficiency for the electrochemical nitrate reduction (NO_3_RR) to produce ammonia. In comparison to pristine MXene, the Cu-entrapped MXene exhibited superior electrochemical nitrate reduction performance. Cu@V_2_C, utilizing V_2_C as the MXene, displayed remarkable efficiency, achieving an 83% faradaic efficiency and ammonia yield of 320 μg cm^–2^ h^–1^ at −0.7 V vs RHE, alongside excellent selectivity and long-term stability. Applying the same methodology to Ti_3_C_2_-derived MXene also yielded substantially improved performance. Although Cu serves as the primary catalytically active component, the electrochemical performance of the catalyst is strongly influenced by the MXene substrate, as evidenced by electrochemical impedance spectroscopy (EIS) analysis. Notably, Cu@V_2_C exhibits superior charge-transfer characteristics at the electrode–electrolyte interface compared to Cu@Ti_3_C_2_, highlighting the critical role of the support in facilitating electron transport and enhancing overall catalytic activity. The results from this project highlight the transformative potential of Cu-entrapped MXenes as next-generation catalysts, synthesized via environmentally friendly methods, for efficient and sustainable nitrate reduction.

## Experimental Section

### Materials

Ti_3_AlC_2_ and V_2_AlC were purchased from Laizhou Kai Kai Ceramic Materials, China. Ethanol (96%) was purchased from Penta Chemicals (Czech Republic). Sodium chloride, potassium chloride, copper chloride, oxalic acid dihydrate, Nafion, sodium sulfate, sodium hydroxide, citric acid, sodium nitroferricyanide, *N*-(1-naphthyl)­ethylenediamine dihydrochloride, sulfanilamide, phosphoric acid, sodium hypochlorite, and salicylic acid were purchased from Merck (Germany). Potassium nitrate was purchased from Alfa Aesar. All the chemicals were analytical grade and used as received. All the solutions were prepared in Millipore water with a resistivity of 18 MΩ cm.

### Synthesis of Cu@V_2_C and Cu@Ti_3_C_2_

Cu@V_2_C was synthesized using the molten salt synthesis (MSS) method in an air atmosphere. In a typical synthesis, an optimized mixture of V_2_AlC MAX phase, anhydrous copper chloride (CuCl_2_), sodium chloride (NaCl), potassium chloride (KCl), and oxalic acid dihydrate was mixed well in a mortar and pestle. Then the mixture was transferred into an alumina crucible with a lid, and the mixture was covered with more NaCl and KCl mixture to avoid air interaction. The crucible with the mixture was heated to 700 °C for 10 h in a muffle furnace. After the reaction, the product was washed repeatedly with deionized (DI) water and ethanol. Finally, the collected powder was dried at 60 °C in a vacuum oven overnight. This sample is named Cu@V_2_C. Cu@Ti_3_C_2_ was synthesized with the same procedure using Ti_3_AlC_2_ MAX, anhydrous copper chloride (CuCl_2_), sodium chloride (NaCl), potassium chloride (KCl), and oxalic acid dihydrate.

### Synthesis of Pure V_2_C and Ti_3_C_2_

The pure MXene samples were synthesized by following the same procedure with precursors, V_2_AlC MAX phase, lithium chloride (LiCl), sodium chloride (NaCl), potassium chloride (KCl), and oxalic acid dihydrate. The vanadium carbide and titanium carbide samples obtained were named as V_2_C and Ti_3_C_2_.

### Physicochemical Characterization

The morphology of MXenes was analyzed by SEM (TESCAN MIRA 3 XMU) at 10 kV, using the carbon tape to mount the samples, and the elemental distribution was studied using an EDS (Oxford Instruments X-MAX20) detector. Chemical composition was analyzed using XPS (Kratos Analytical Axis Supra) equipped with a monochromatic Al Kα X-ray source (1486.6 eV). The synthesized Cu@MXene-powder samples were mounted onto a double-sided copper tape. Charge neutralization was achieved using an electron flood source operated around 3.5 eV. The survey spectra were recorded with a pass energy of 80 eV and a step size of 1 eV. All spectra were calibrated against the C 1s peak of adventitious carbon at 284.8 eV and analyzed using Casaxps software. XRD patterns were collected using an X-ray diffractometer (Rigaku SmartLab 3 kW) in Bragg–Brentano geometry with a Cu Kα radiation source. All the UV–visible absorbance was performed by a UV–visible spectrometer (JASCO V-750). The proton NMR measurement was carried out using a Bruker Advance (700 Hz) and analyzed using Topspin 4.1.1 software.

### Electrochemical Measurement

All electrochemical measurements were performed using a potentiostat (T204, Metrohm Autolab, Netherlands) connected to a computer and operated with NOVA software (version 2.1). A three-electrode configuration was employed with Pt as the counter electrode, Ag/AgCl as the reference electrode, and electrocatalyst-coated glassy carbon as the working electrode. The Cu@MXenes were made into a suspension in a 1:1 ethanol–water mixture and 5 wt % Nafion (4 mg mL^–1^), followed by sonication in a bath sonicator for 20 min. The sonicated MXenes were coated (30 μL) on the glassy carbon electrode, allowed to dry in the air, and used as the working electrode.

The electrochemical impedance spectroscopy was measured in 0.5 M Na_2_SO_4_ + 0.1 M KNO_3_ in the frequency range of 100 kHz to 0.1 Hz with an amplitude of 20 mV. The data is analyzed using EC-lab V11.33 software with the Randomize and Normalize iteration model. The linear sweep voltammetry was carried out in 0.5 M Na_2_SO_4_ solution and 0.5 M Na_2_SO_4_ + 0.1 M KNO_3_ with a scan rate of 5 mVs^–1^ to study the nitrate reduction activity. An H-cell separated by a glass-frit was used to carry out the electrolysis, distributing the electrolyte evenly on both anodic and cathodic chambers (20 mL each). The electrolysis was performed for 1 h for every sample with different applied potentials. The solution was kept under continuous stirring at 175 rpm to study the ammonia yield and FE. All the potential was converted into a reversible hydrogen electrode (RHE).

The electrochemically active surface area was estimated using a cyclic voltammogram of catalysts in 3 M NaOH in a nonfaradaic voltage range of 0 to 0.6 V, utilizing eq 1

1 $$\mathrm{ECSA}=C_{S}/C_{dl}\;\left({\mathrm{cm}}^{2}g^{-1}\right)$$

The *C*_dl_ of the cell was estimated using the CV curves at different scan rates (20, 40, 60, 80, and 100 mV s^–1^). The *j*_a_ (anodic current density) and *j*_c_ (cathodic current density) were taken at 0.3 V. Then the *C*_dl_ (F cm^–2^) was taken from the slope of the curve Δj2 (where $\Delta j=\frac{\left|j_{a}-j_{c}\right|}{2}$ ) versus scan rate. *C*_S_ (Fg^–1^) is taken as the specific capacitance at scan rates, where $C_{S}=\frac{\mathrm{Area under the CV}}{\mathrm{active mass}\times \mathrm{scan rate}\times \mathrm{potential window}}$ .

### Quantification of Ammonia Production

After each electrolysis, the electrolytes were collected and analyzed using a UV–visible spectrophotometer (JASCO V-750) to quantify the ammonia production. A portion of the electrolyte was taken and diluted up to 600 μL, followed by the addition of a reagent solution containing 3 M NaOH, 10 wt % salicylic acid, and 10 wt % citric acid. Subsequently, 300 μL of a second solution containing 0.2 M NaClO and 60 μL of 2.0 wt % sodium nitroferricyanide was added, and the mixture was allowed to rest for 2 h. The ammonia concentration was determined from the indophenol blue product by measuring the absorbance at 655 nm wavelength and calculations based on the calibration curve (Figure S5) using a series of standard NH_4_Cl solutions with 0.5 M Na_2_SO_4_.^34^ The FE and YR were then calculated using the following equations:

2 $$\mathrm{FE}=\left(n\times F\times c_{NH_{3}}\times V\right)/Q$$

3 $$\mathrm{YR}=\left(c_{NH_{3}}\times V\right)\times 17/\left(t\times S\right)$$

Where *n* is the number of electrons involved in the chemical reaction, 8 and 2, respectively, for ammonia and nitrite reduction, *F* is the Faradaic constant (96485 C mol^–1^), $c_{NH_{3}}$ is the concentration of measured NH_3_, *V* is the electrolyte volume of the cathodic compartment, *Q* is the total charge passing the electrode, *t* is the electrolysis time, and *S* is the surface area of the working electrode.

### Quantification of Nitrite

The nitrite (NO_2_^–^) quantification was carried out using a previously reported protocol using the Griess test. The color reagent (Griess reagent) was prepared by adding *N*-(1-naphthyl) ethylenediamine dihydrochloride (0.02 g), sulfanilamide (0.4 g), ultrapure water (5 mL), and phosphoric acid (1 mL, d = 1.70 g). The electrolyte collected after the electrolysis is taken out and diluted to an appropriate dilution range up to 1.5 mL, then 50 μL of coloring reagent is added and rested for 20 min, in which the sulfonamide reacts with the NO_2_^–^ to form a diazonium salt and then further reacts with the amine to form an azo dye (magenta). Ultraviolet–visible spectroscopy measured the absorbance at 540 nm, and the NO_2_^–^ concentration was calculated. The NO_2_^–^ concentration was calculated based on the calibration curve (Figure S6), which was obtained by a series of standard NaNO_2_ solutions with 0.5 M Na_2_SO_4_.

### Determination of Ammonia by ^1^H NMR (Isotopic Labeling Experiment)

For the isotopic labeling, we carried out the electrolysis using 0.1 M K^15^NO_3_ for 1 h, and 5 mL of the electrolyte was collected after the electrolysis. 250 μL of concentrated sulfuric acid is added to the solution to adjust the pH value. Followed by the addition of 0.002 g Maleic acid as the internal standard. 500 μL of the as-prepared solution is added to an NMR tube and 50 μL of deuterium oxide (D_2_O) is also added for NMR detection.

## Supplementary Material



## Notes

XPS wide spectra of Cu-infused V_2_C (Cu@V_2_C) (Figure S1); charge transfer properties of Cu@V_2_C (Figure S2); H-cell set up used for electrochemical ammonia synthesis (Figure S3); electrolysis using pure V_2_C and ammonia detection (Figure S4); ammonium and nitrite ion detection formed using Cu@V_2_C catalyst (Figure S5); calibration curve for ammonia (Figure S6); ECSA for Cu@V_2_C (Figure S7); nitrite calibration curve (Figure S8); SEM image after 10 cycles of reaction (Figure S9); EDS image of Cu@Ti_3_C_2_ (Figure S10); XPS of Cu@Ti_3_C_2_ (Figure S11); charge transfer properties for Cu@Ti_3_C_2_ (Figure S12); electrolysis and ammonia detection using Ti_3_C_2_ (Figure S13); electrolysis and ammonia detection using Cu@Ti_3_C_2_ (Figure S14); ECSA for Cu@Ti_3_C_2_ (Figure S15); detection of nitrite ion formed using Cu@Ti_3_C_2_ (Figure S16); the durability tests of Cu@Ti_3_C2 (Figure S17) (PDF)
