# Supplementary material for: Copper-Infused MXene from MAX Phase for Enhanced Electrochemical Ammonia Production
Source: ACS Nano. 2025 Nov 12;19(46):39645–53. doi: 10.1021/acsnano.5c09659 (PMC12659423; doi:10.1021/acsnano.5c09659)
Supplement: Supplementary file 1 [file nn5c09659_si_001.pdf]

Copper-Infused MXene from MAX Phase for Enhanced Electrochemical Ammonia Production

Radhika Nittoor-Veedu[a,b], *Bindu Kalleshappa*[a], *Martin Pumera**[a,b,c,d,e,f]

[a] Future Energy and Innovation Laboratory, Central European Institute of Technology, Brno University of Technology, Purkyňova 123, Brno, 61200, Czech Republic

[b] Quantum Materials Laboratory, 3D Printing and Innovation Hub, Center for Nanorobotics and Machine Intelligence, Department of Chemistry and Biochemistry, Mendel University, Zemědělská 1, Brno, 61300, Czech Republic

[c] Department of Medical Research, China Medical University Hospital, China Medical University, No. 91 Hsueh-Shih Road, Taichung, 40402, Taiwan

[d] Advanced Nanorobots & Multiscale Robotics Laboratory, Faculty of Electrical Engineering and Computer Science, VSB - Technical University of Ostrava, 17. listopadu 2172/15, 70800 Ostrava, Czech Republic

[e] Department of Chemical and Biomolecular Engineering, Yonsei University, 50 Yonsei-ro, Seodaemun-gu, Seoul, 03722, Korea

[f] Energy Research Institute@NTU (ERI@N), Research Techno Plaza, X-Frontier Block, Level 5, 50 Nanyang Drive, 637553 Singapore, Singapore

Corresponding author email: martin.pumera@gmail.com

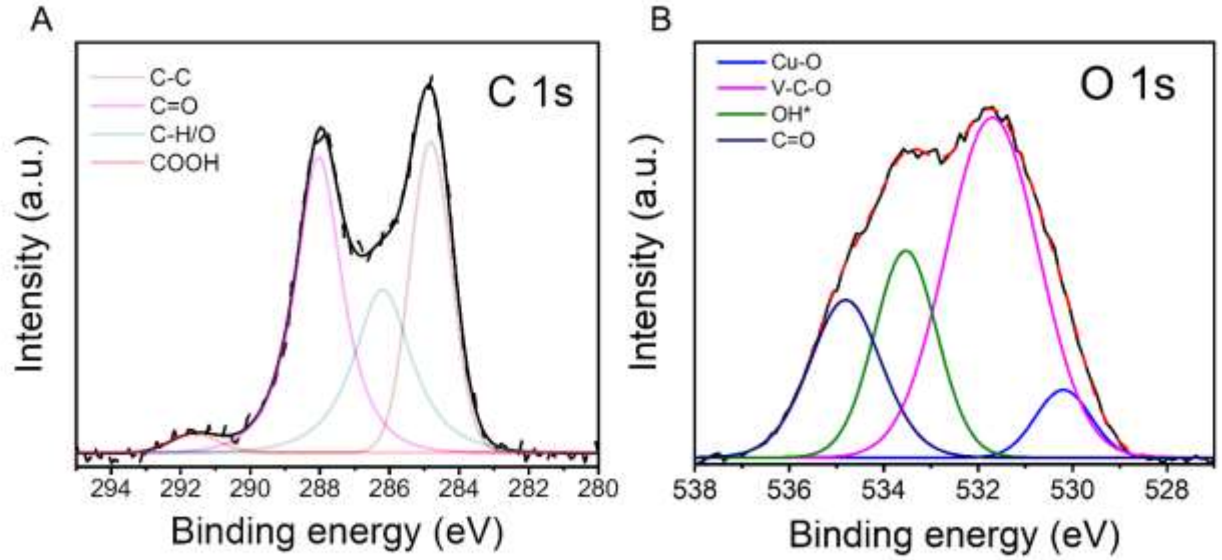

Figure S1: XPS Cu@V_2C **A)** C 1s **B)** O 1s.

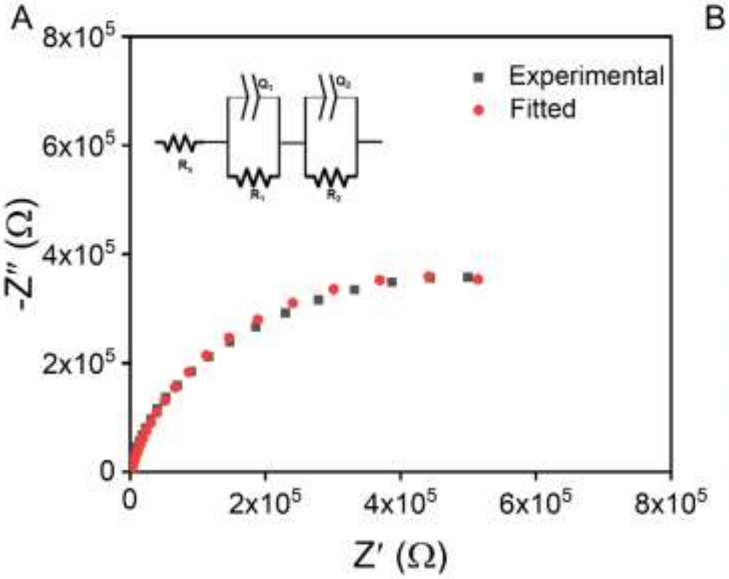

B

Factors	Values
R_s	1116 Ω
R_1	174 k Ω
Q_1	8.53 mS.s^n
n_1	0.82
R_2	778 kΩ
Q_2	0.27 mS.s^n
n_2	0.87

Figure S2. A) Fitted Nyquist plot of Cu@V_2C with the equivalent circuit (inset), and its **B)** parameters.

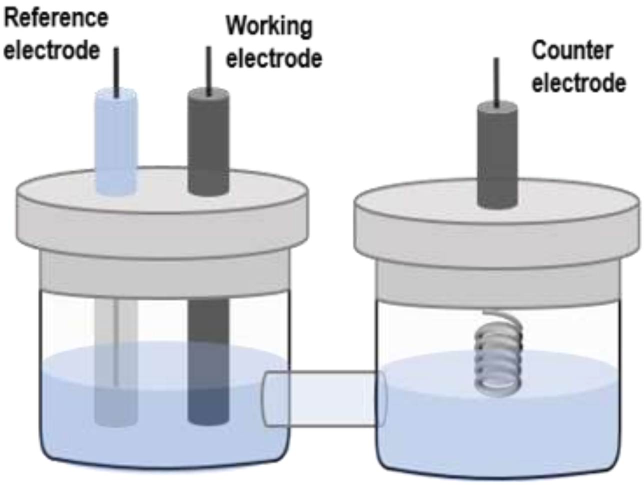

Figure S3. H cell setup used for electrolysis

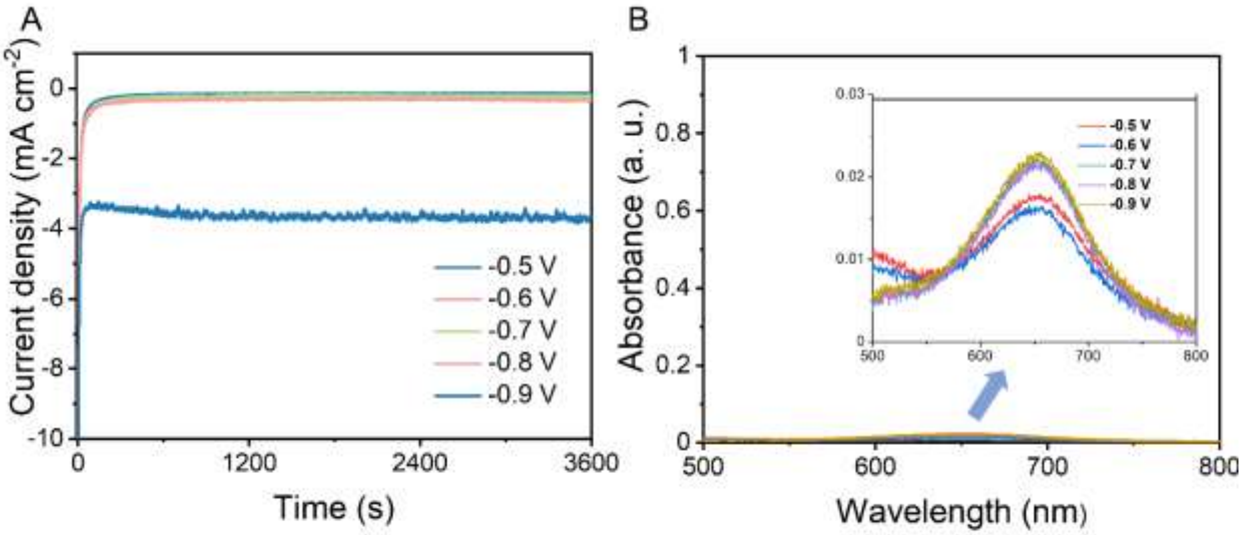

Figure S4. A) Chronoamperometry curves for V_2C at different potentials. **B)** UV-Vis absorption spectra of electrolysis solution at different potentials.

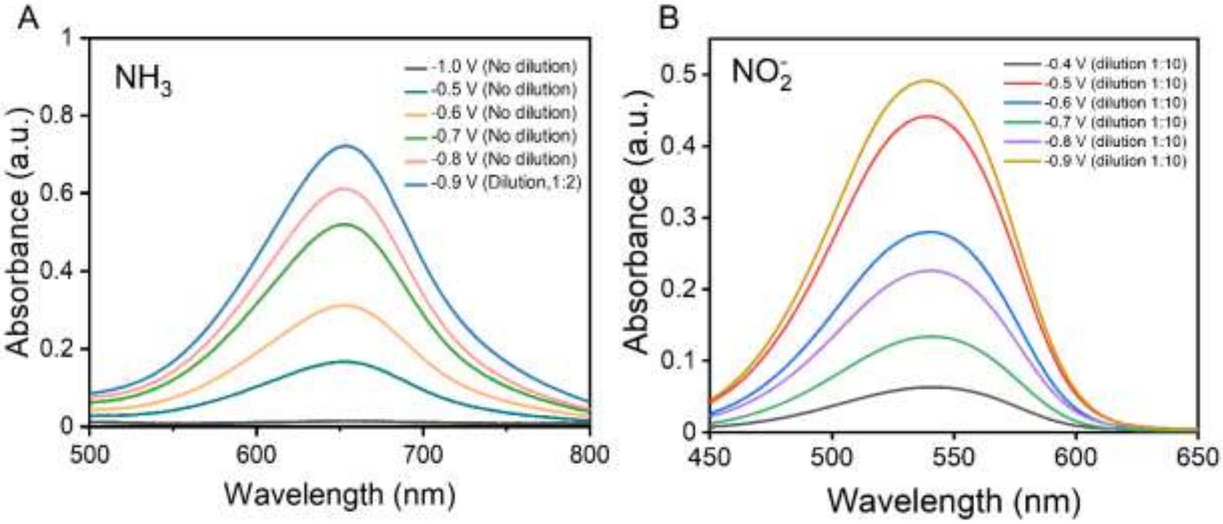

Figure S5. UV-Vis absorbance spectra of Cu@V_2C for **A)** Ammonium ions and **B)** nitrite ions at different applied potentials.

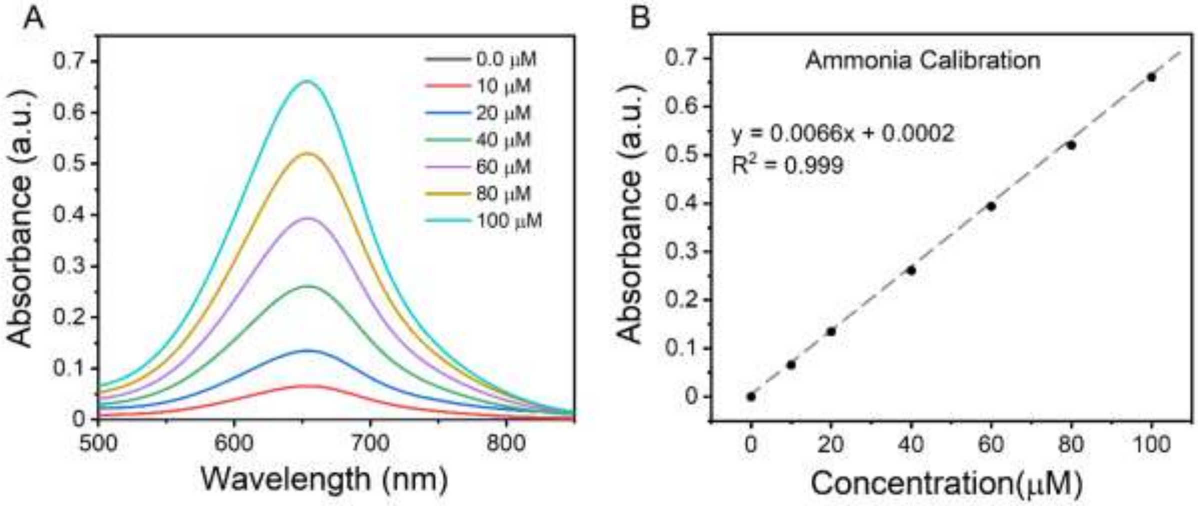

Figure S6. **A)** UV-Vis absorbance spectra using standard solutions of NH_4Cl. **B)** Ammonia calibration curve.

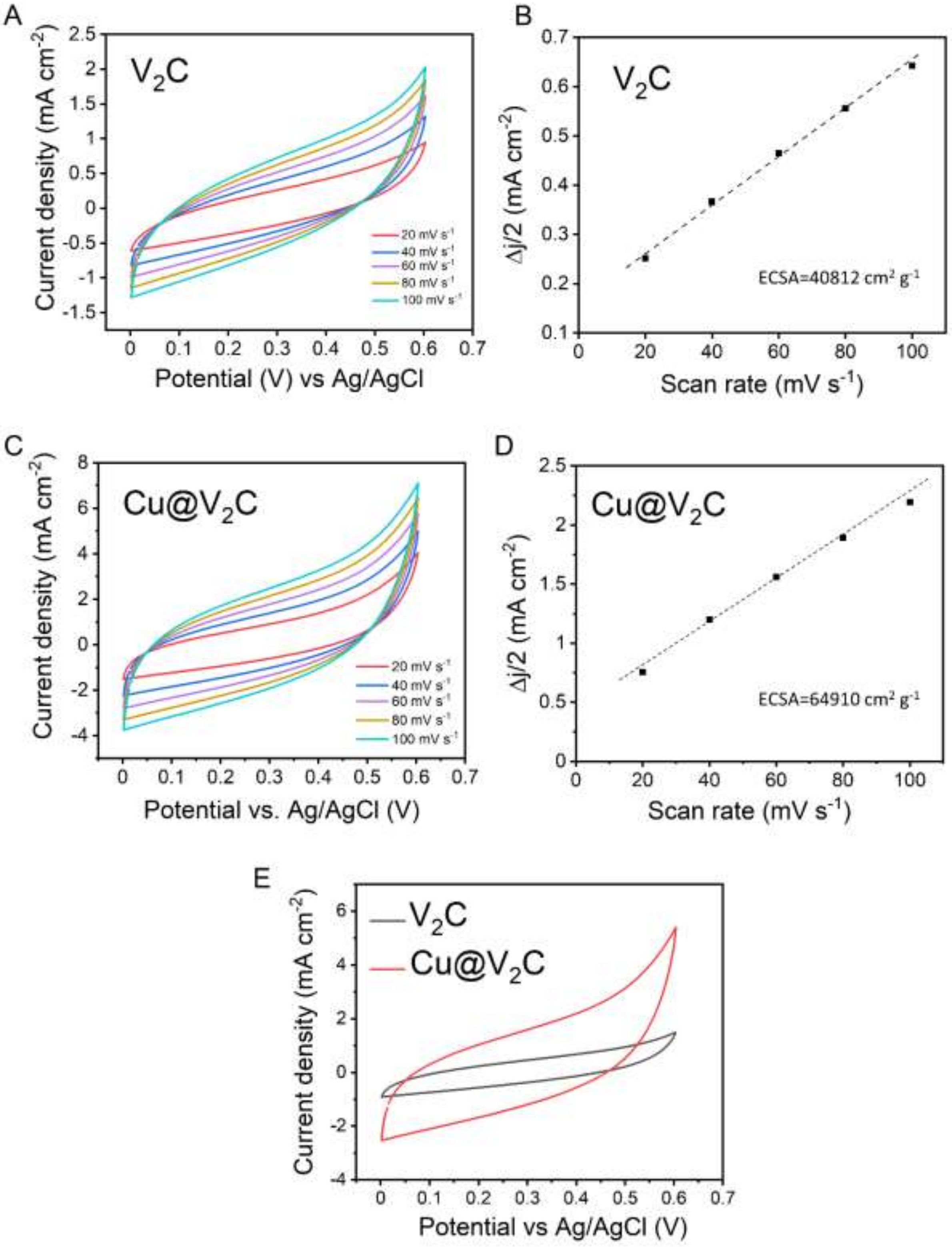

Figure S7. A) Cyclic voltamogram at non-faradaic potential for V_2C with different scan rates. **B)** Difference between anodic and cathodic current values at 0.3 V vs. Ag/AgCl for V_2C, at

different scan rates. **C)** Cyclic voltamogram at non-faradaic potential for Cu@V_2C with different scan rates. **D)** Difference between anodic and cathodic current values at 0.3 V vs. Ag/AgCl for Cu@V_2C, at different scan rates. **E)** Comparison between V_2C and Cu@V_2C at 10 mV s^{-1} scan rate.

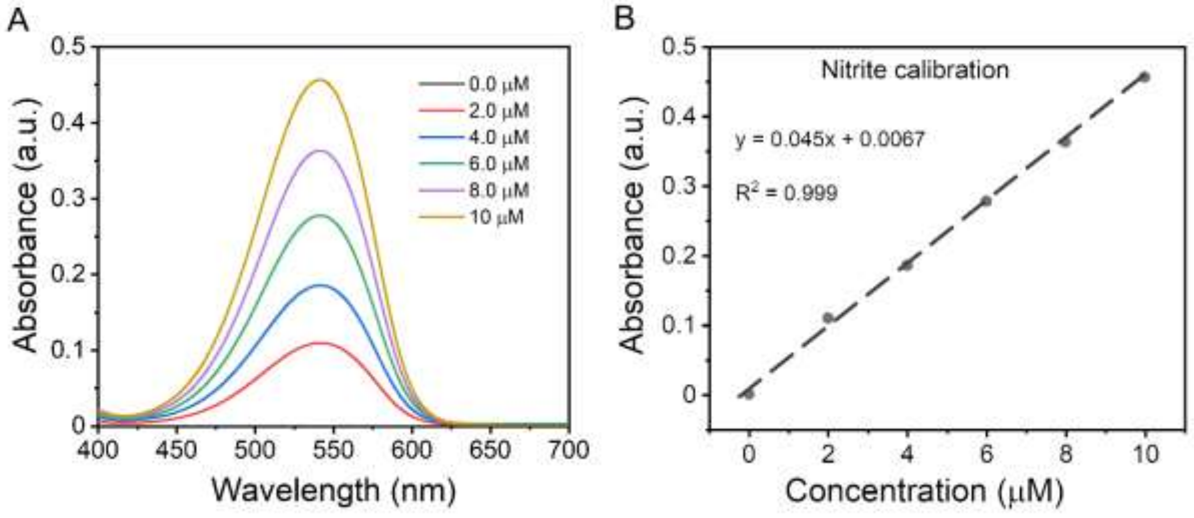

Figure S8. **A)** UV-Vis absorbance spectra using standard solutions of $NaNO_2$. **B)** Nitrite calibration curve

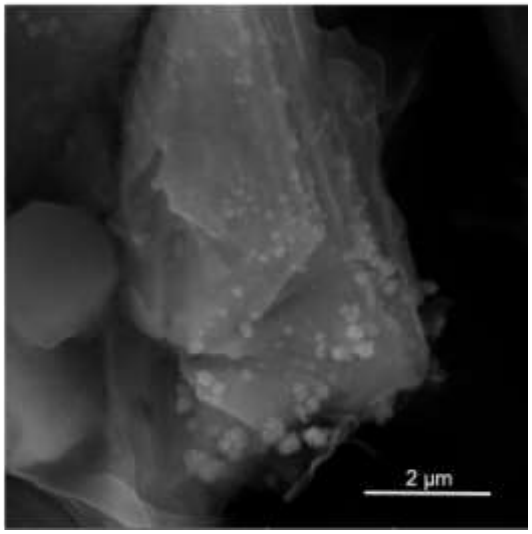

Figure S9. SEM micrograph of Cu@V_2C after 10 cycles

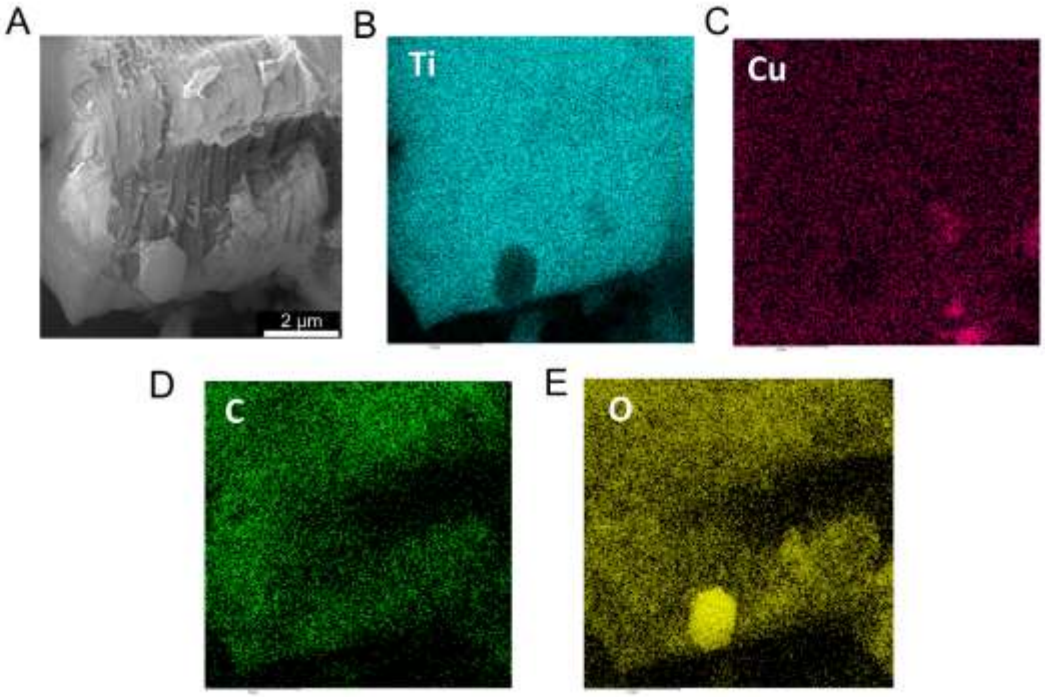

Figure S10. Elemental distribution on $Cu@Ti_3C_2$. **A)** scanning electrochemical microscope image of $Cu@Ti_3C_2$ **B)** Ti, **C)** Cu, **D)** C, and **E)** O

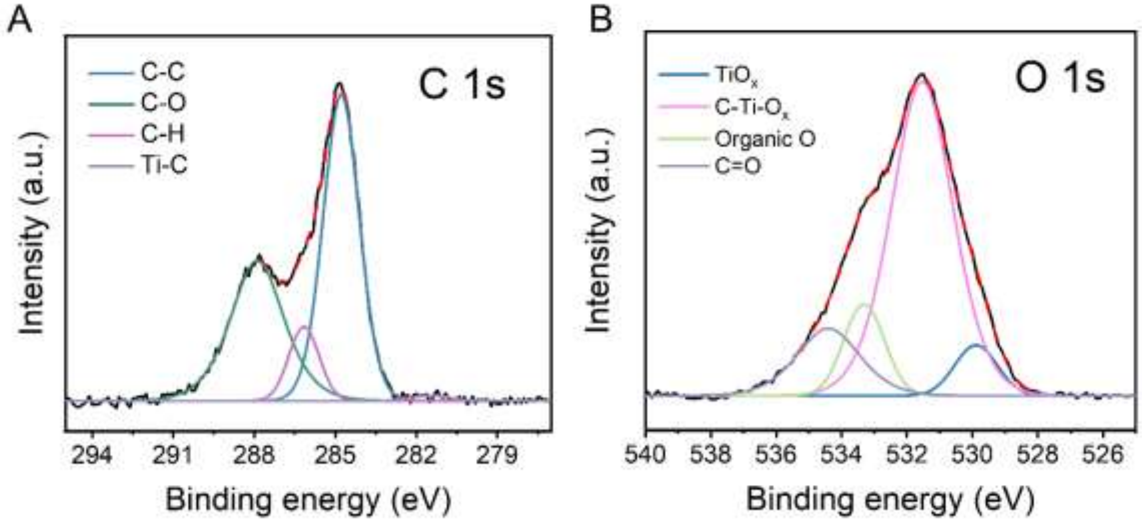

Figure S11. High resolution spectra of $Cu@Ti_3C_2$ **A)** C 1s, **B)** O 1s

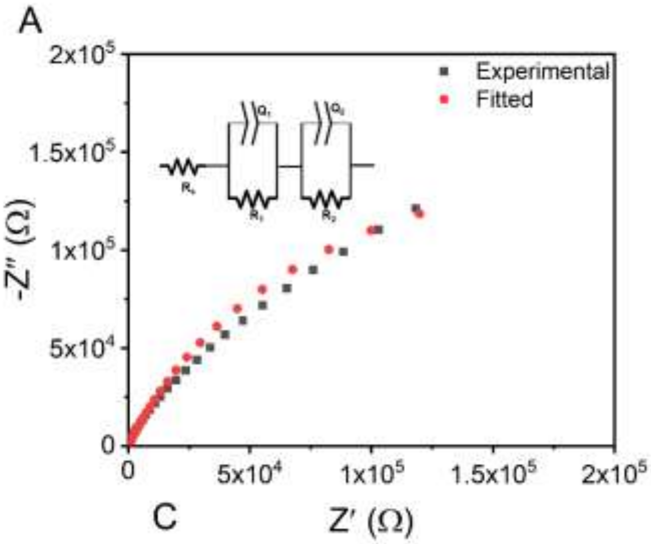

B

Factors	Values
R_s	114 Ω
R_1	1324 k Ω
Q_1	5.31 mS.s^n
n_1	0.79
R_2	110 kΩ
Q_2	1.92 mS.s^n
n_2	0.80

Figure S12. A) Fitted Nyquist plot of Cu@Ti_3C_2 with the equivalent circuit (inset), and its **B)** parameters.

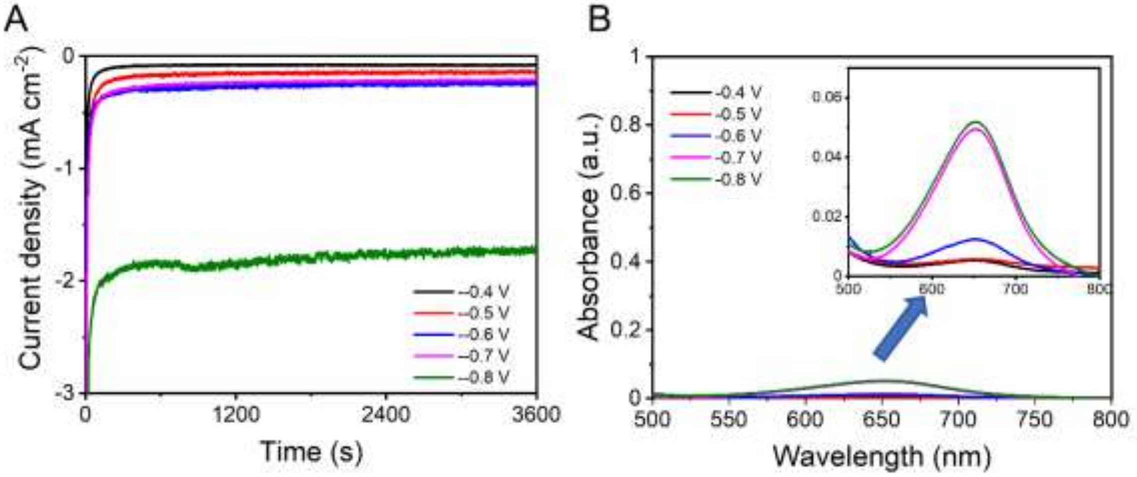

Figure S13. A) Chronoamperometry curves for Ti_3C_2 at different potentials. **B)** UV-Vis absorption spectra of electrolysis solution at different potentials.

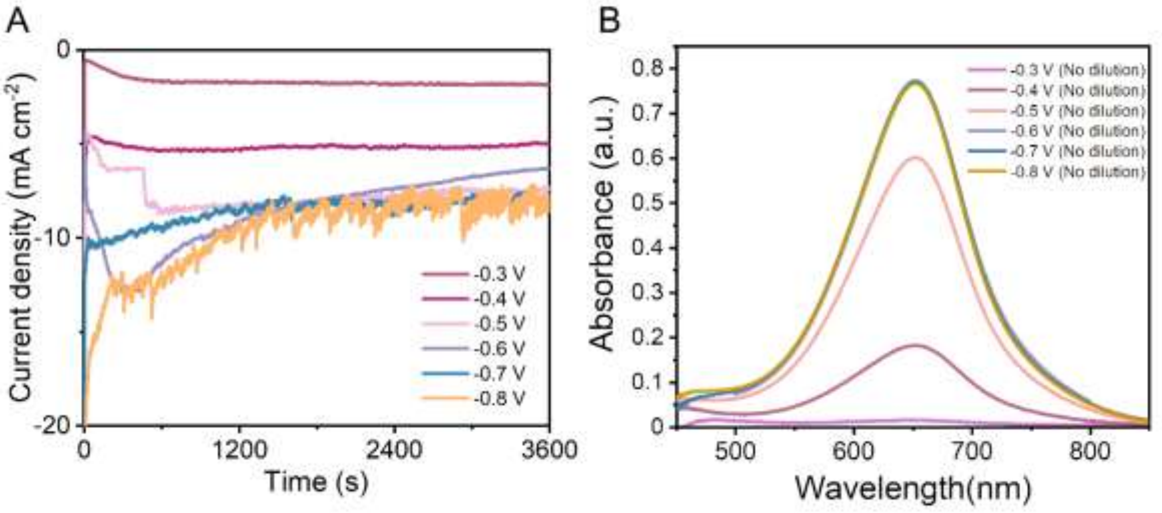

Figure S14. A) Chronoamperometry curves for Cu@Ti_3C_2 at different potentials. **B)** UV-Vis absorption spectra of electrolysis solution at different potentials.

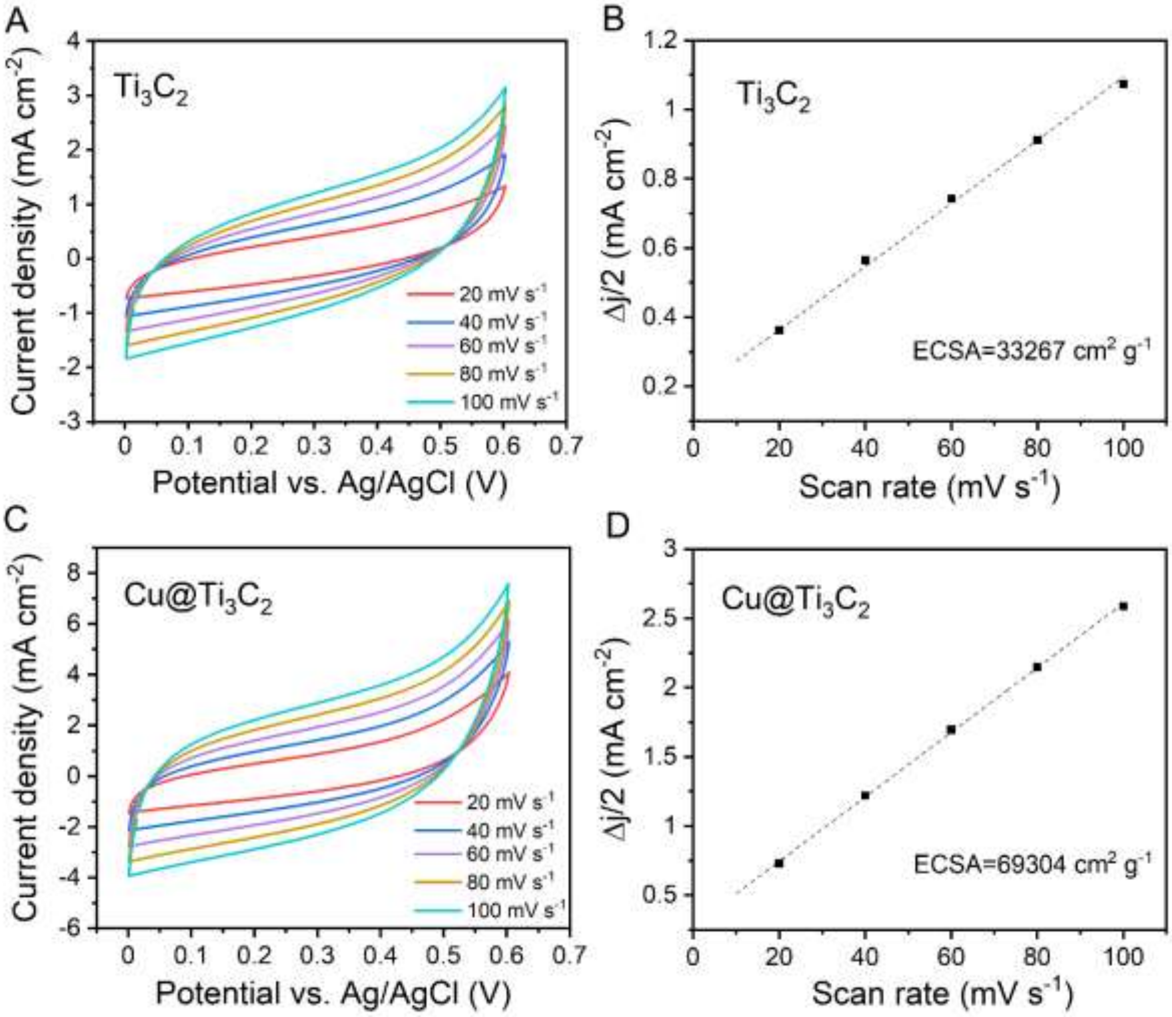

Figure S15. A) Cyclic voltamogram of Ti_3C_2 with different scan rates. **B)** Difference between anodic and cathodic current values at 0.3 V vs. Ag/AgCl for Ti_3C_2, at different scan rates. **C)** Cyclic voltamogram of $Cu@Ti_3C_2$ with different scan rates. **D)** Difference between anodic and cathodic current values at 0.3 V vs. Ag/AgCl for $Cu@Ti_3C_2$, at different scan rates.

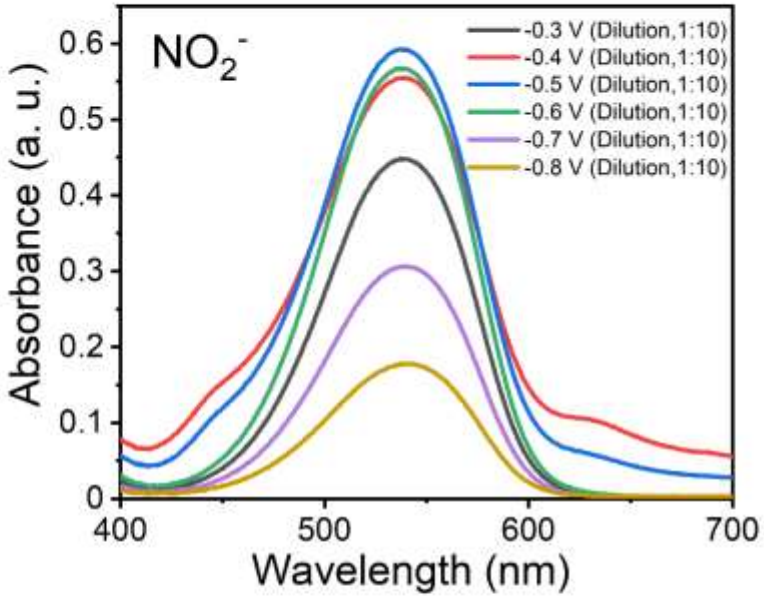

Figure S16. UV-Vis absorbance spectra of Cu@Ti_3C_2 for nitrite ion at different applied potentials.

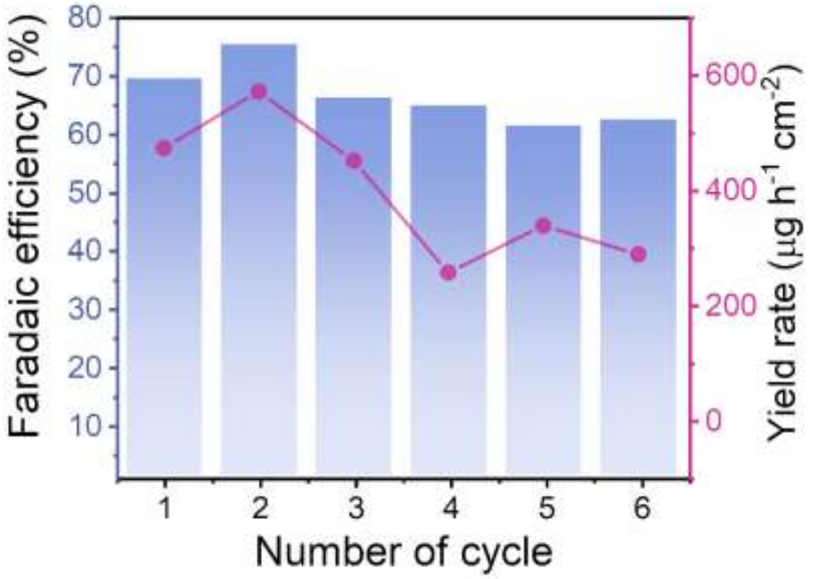

Figure S17. The durability tests of Cu@Ti_3C_2 for NO_3RR at − 0.6 V vs. RHE.
